# Proposed New Definitions of Practice Guidelines for Health and Health Care

**DOI:** 10.31662/jmaj.2025-0066

**Published:** 2025-08-29

**Authors:** Toshio Morizane, Fujimi Kawai, Noriko Kojimahara

**Affiliations:** 1 Department of Public Health, Shizuoka Graduate University of Public Health, Shizuoka, Japan

**Keywords:** background question, clinical practice guideline, clinical question, guidance, health care guideline, health care question, health guidelines, health question, systematic review

## Abstract

Clinical practice guidelines (CPGs) are traditionally defined as systematically developed recommendations designed to optimize patient care, primarily within clinical settings under physician supervision. However, the scope of health-related decision-making has expanded considerably, now encompassing interventions implemented by various health professionals and even the general public. This evolving landscape necessitates a broader and more nuanced classification of health-related guidelines.

In this opinion paper, we propose a revised framework that categorizes guidelines into three distinct types: CPGs, health care guidelines (HcGs), and health guidelines (HGs). CPGs remain focused on physician-led care but may include health-related practices outside clinical settings when relevant to patient outcomes. HcGs address evidence-based practices conducted by licensed non-physician professionals such as nurses, pharmacists, and physical therapists. HGs pertain to practices adopted by the general public―such as diet, exercise, and wellness behaviors―that do not require professional supervision.

We also differentiate between “guidelines,” which provide comprehensive recommendations across a broad range of topics, and “guidance,” which offers targeted recommendations on specific issues. Additionally, we discuss “consensus statements” as a valid alternative when systematic reviews are not feasible.

By clarifying terminology and aligning guideline types with their intended audiences and settings, this framework aims to reduce confusion, improve usability, and promote evidence-based practices across the health care and public spectrum. Adoption of this classification supports a whole-of-society approach to health, aligning with the World Health Organization’s vision for integrated and equitable primary health care systems.

## Introduction

The Institute of Medicine (IOM) ^(1)^ defined clinical practice guidelines (CPGs) in 2011 as: “Clinical practice guidelines are statements that include recommendations intended to optimize patient care that are informed by systematic reviews of evidence and assessments of the benefits and harms of alternative care options.” Similar definitions have been adopted by organizations such as the GRADE (Grading of Recommendations, Assessment, Development and Evaluation) ^(2)^ working group, the National Institute for Health and Care Excellence, and Japan’s Medical Information Network Distribution Service ^(3)^.

As the term suggests, CPGs primarily focus on medical practices performed by physicians in clinical settings. They are intended to facilitate shared decision-making between health care providers and patients. However, health-related decision-making today extends far beyond the clinical environment. Increasingly, interventions relevant to health are being made by other health care professionals―and even by the general public. This evolution aligns with the World Health Organization’s ^(4)^ concept of primary health care, which promotes a whole-of-society approach to strengthening global health systems. Accordingly, multisectoral policies and interventions must address the broader determinants of health. Recommendations in all these domains should ideally be grounded in scientific evidence, typically derived from epidemiological studies and synthesized through systematic reviews ^(5), (6)^. In this opinion paper, we propose updated definitions for “guidelines,” “guidance,” and “consensus statements” to better reflect the expanding scope of health-related decision-making.

## Framing the Questions

Formulating clear questions is essential for identifying relevant evidence from databases such as PubMed, Embase, Cochrane CENTRAL, and CINAHL. The PICO framework―Population, Intervention, Comparator, Outcomes―supports targeted searches and informed study selection. While “Clinical Questions (CQs)” are standard in evidence-based medicine and CPGs, we propose the additional categories of “Health Care Questions (HcQs)” and “Health Questions (HQs)” for guidelines addressing broader health domains. Collectively, we refer to these as “questions.” These typically fall into two types: Foreground Questions (FQs), which focus on specific clinical decisions ^(7)^ and support evidence-based recommendations; and background questions (BQs), which explore broader contextual information. Both types are critical to the structure and utility of guidelines. For example, understanding the range of effective options (BQ) is essential before choosing among them (FQ).

## Three Categories of Guidelines

**Figure 1.**
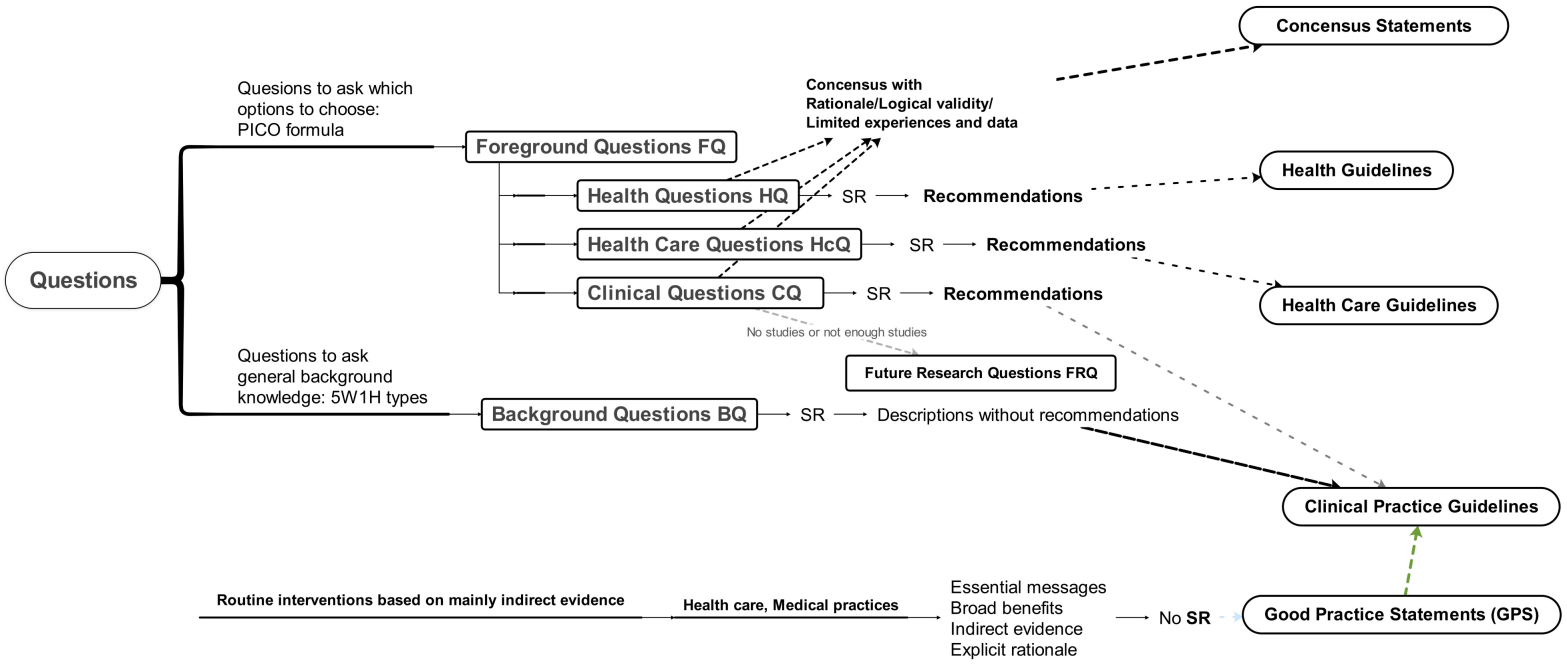
When the topic is limited “guidance” should be used instead of “guidelines.” “Clinical Practice Guidelines” can include “Health Care Guidelines” and “Health Guidelines.” Health care is provided by qualified health care professionals, and medical practices are delivered only by qualified physicians. SR for the BQ is not compulsory. . 5W1H: When, Where, Who, What, Why, How; BQ: background question; PICO: Population, Intervention. Comparator, Outcome, SR: systematic review.

A PubMed search conducted on April 23, 2025, using the MeSH term “practice guidelines” returned 33,622 results. In contrast, only seven results mentioned “health care guidelines,” 17 included “health guidelines,” and 3,504 referenced “clinical practice guidelines.” This suggests that most health-related recommendations are published under the term “clinical practice guidelines,” even when the intended audiences or contexts differ. To reduce ambiguity and improve the usability of such guidelines―particularly for non-physician professionals and the general public, we propose categorizing them into three groups shown in Figure 1.

### Health guidelines

Health Guidelines (HGs) address health-related practices outside the formal health care system that do not require physician involvement or institutional care. Examples include exercise, diet, and wellness apps. While these guidelines may be overseen by regulatory bodies (e.g., Japan’s Ministry of Health, Labor and Welfare), they are typically not integrated into clinical care. For instance, dietary recommendations developed using the GRADE approach have been published in medical journals ^(8)^ but they primarily target the general population. To enhance clarity and reach, such documents should be labeled “Health Guidelines,” and systematic reviews are recommended for their development.

### Health care guidelines

Health Care Guidelines (HcGs) focus on medical practices conducted by licensed professionals such as nurses, pharmacists, and physical therapists. Some of these practices require physician oversight, while others fall within the independent scope of the practitioner. A relevant example is the guideline titled “Clinical Practice Guidelines for Assessment of Colonic Retention in Constipation for Nursing Care,” published by the Japan Academy of Nursing Science ^(9)^. These guidelines belong within the health care domain and are subject to legal regulation.

### Clinical practice guidelines

CPGs traditionally refer to care provided by physicians or under their supervision. However, modern CPGs may include elements beyond the clinical setting―such as diet or exercise―when these are essential to patient care. CPGs may incorporate HcQs or HQs to support a holistic approach. They may also feature “Good Practice Statements (GPS),” which present widely accepted practices based on indirect evidence or strong rationale. Because GPS are grounded in expert consensus rather than direct evidence, systematic reviews are not required.

## Guidelines vs. Guidance

“Guidance” refers to brief recommendations focused on a specific topic or a narrow set of questions. These are suitable when the issue can be addressed independently and the recommendation is clear-cut. In contrast, “Guidelines” are more comprehensive, often covering the entire continuum of a disease or intervention, including diagnostic, therapeutic, and preventive strategies.

## Consensus Statements

When systematic reviews are not feasible but recommendations are still needed, developers may issue consensus statements. These rely on expert consensus, logical reasoning, and limited available data. In some cases, systematic reviews may be included, though they are not required. Consensus statements serve as a practical alternative when a full evidence synthesis is impractical. Documents labeled “handbook” or “guide” may also summarize existing guidelines for wider audiences. However, these labels should not be used as a means to bypass the rigorous methodology required for developing evidence-based recommendations.

The widespread use of “clinical practice guidelines” for all types of health-related recommendations contributes to confusion. Guidelines aimed at the general public are often underused, as the term “clinical” implies they are exclusively for health care professionals. Similarly, guidelines for non-physician professionals can be difficult to apply due to physician-centered formatting, even when created collaboratively. While health care practices differ globally, most countries require professional licensure for individuals who deliver care. Services may be divided into physician-only, physician-supervised, or independently practiced care by other licensed professionals. Simultaneously, many health-related decisions―such as dietary choices, exercise routines, supplement use, and engagement with digital health tools―are made by individuals outside the formal health care system.

### Conclusion

We propose the formal adoption of three distinct categories―CPG, HcG, and HG―to more accurately represent the diversity of modern health-related recommendations. This classification aims to clarify target audiences, enhance accessibility, and encourage a more evidence-based approach to health practices in both clinical and non-clinical settings. Standardizing terminology and expectations will help strengthen global health systems and support informed individual health decisions.

## Article Information

### Conflicts of Interest

None

### Acknowledgement

We thank the members of the AMED Guidelines for Digital Health Technology Prevention Intervention for Mental Health for their helpful discussions on the development of health guidelines.

### Author Contributions

Conception of the work: Toshio Morizane , Fujimi Kawai , and Noriko Kojimahara. Writing of the paper: Toshio Morizane. Critical revision of the paper: Fujimi Kawai , and Noriko Kojimahara. All authors have read the final draft and approved it for submission.
